# Degumming of Ramie Bast Fibers by *Pectobacterium carotovorum* HG-49: Mechanisms and High-Efficiency Strategies

**DOI:** 10.3390/polym18141775

**Published:** 2026-07-20

**Authors:** Tong Shu, Tianyi Yu, Pandeng Li, Ziqi Hou, Huihui Wang, Yulong Chen, Chunhua Fu, Longjiang Yu

**Affiliations:** 1 Institute of Resource Biology and Biotechnology, Department of Biotechnology, College of Life Science and Technology, Huazhong University of Science and Technology, Wuhan 430074, China; shutong0618@126.com (T.S.); yu_tianyi@hust.edu.cn (T.Y.); lipandeng@hust.edu.cn (P.L.); ziqi_h@163.com (Z.H.); yl_chen@hust.edu.cn (Y.C.); 2 Key Laboratory of Molecular Biophysics, Ministry of Education, Wuhan 430074, China; 3 School of Laboratory Medicine, Hubei University of Chinese Medicine, Wuhan 430074, China; huihuiwang@hbucm.edu.cn

**Keywords:** ramie fibers, microbial degumming, degumming process, degumming mechanism, high-efficiency strategy

## Abstract

Microbial degumming offers an eco-friendly alternative to chemical methods for ramie fiber production, but industrial application is constrained by low efficiency stemming from limited mechanistic insight. This study systematically investigates the process using *Pectobacterium carotovorum* HG-49. Strain HG-49 showed a lag phase of 0–4 h, a logarithmic phase of 6–10 h, and peak biomass at 12 h. Pectin (97.05%) and water-soluble substances (98.45%) were nearly fully removed, whereas hemicellulose removal was only 73.54%, rendering it the primary residual gum component. Pectinase activity peaked at 120.75 U/mL, while mannanase (35.85 U/mL) and xylanase (30.20 U/mL) reached roughly one-quarter of that level; cellulase activity remained minimal. Scanning electron microscopy (SEM) indicated that 6–12 h constituted the main gum degradation phase. Fourier transform infrared spectroscopy (FTIR) and micro-FTIR showed progressive decreases in pectin, hemicellulose, and lignin absorption peaks with degumming. X-ray diffraction (XRD) revealed increased crystallinity from 72.07% to 80.02%, and thermogravimetric analysis (TGA) showed elevated degradation temperature from 417 °C to 435 °C. Collectively, these data confirm progressive removal of gummy substances and enhanced cellulose purity. Transcriptomic profiling further revealed that low abundance and reduced expression of hemicellulases significantly limited degumming performance. Therefore, enhancing efficiency should focus on: supplementing pectin-rich substrates to accelerate bacterial proliferation and enzyme production, broadening the hemicellulase spectrum and enhancing catalytic activities and establishing effective pretreatment protocols for ramie bast. These findings provide a theoretical foundation for improving microbial degumming efficiency and advancing industrial feasibility.

## 1. Introduction

With growing public concerns for the environment and resource, natural fibers are becoming increasingly popular due to their biodegradable and renewable properties [1,2]. Ramie fiber, renowned as the “King of Natural Fibers,” exhibits outstanding properties such as high tenacity, a silk-like luster, antibacterial effects, and abrasion resistance [3,4]. This makes it suitable for a wide range of applications, including textiles, clothing, packaging, automotive interiors, medical gauze, and military ropes [5]. Ramie bast fibers comprise approximately 70% cellulose, along with about 30% gummy components, primarily pectin, hemicellulose, and water-soluble substances. These impurities tightly enveloped the cellulose and must be removed through degumming prior to spinning, so as to obtain cellulosic fibers with good spinnability [6]. Degumming is the most critical step for enabling the practical application of ramie fibers. Conventional chemical degumming relies on the extensive use of sulfuric acid and alkaline solutions to eliminate non-cellulosic gums from the fibers, and it is highly polluting and energy-intensive, which does not align with the requirements of green development [7]. Consequently, biological degumming has steadily risen to prominence as the preferred approach, owing to its environmentally sustainable properties [8,9].

Biological degumming primarily consists of microbial and enzymatic methods [10]. However, due to the complex composition nature of the gum materials from ramie bast, effective results require the synergistic action of multiple enzymes, which makes enzymatic degumming process complex and costly [11]. Microorganisms possess a wide variety of enzyme systems, thereby offering simple operation, low cost and good performance [9]. However, this approach is time-consuming, often requiring several days to over 10 days to complete the process. Our research group previously discovered a *Pectobacterium carotovorum* HG-49 strain, with outstanding degumming capability for ramie bast fibers [12]. It removes 82.16% of gums within 16 h, which represents a leading level among reported studies [13,14,15]. Although the high efficiency already achieved, a considerable gap still exists between the current performance and practical industrial application. To better realize its potential for industrial application, increasing its degumming efficiency is an urgent necessity [4].

Improving microbial degumming efficiency requires a thorough understanding of the process itself [16]. Microbial degumming is a systematic process involving the interaction of multiple factors, including the growth and reproduction of degumming strains, the expression and secretion of degumming enzymes, the degradation and utilization of gums, changes in the physicochemical conditions of the degumming environment, and changes in the morphology and structure of ramie fibers. All the above factors may have a significant impact on the microbial degumming efficiency. However, current research on microbial degumming has mainly focused on strain screening [13,14], optimization of degumming conditions [17], pretreatment of ramie bast [18,19], combination of different degumming strains [15], and engineering strain construction [20]. There has been almost no comprehensive or systematic research on the microbial degumming process itself. As a result, current studies have made very limited progress in improving microbial degumming efficiency.

To significantly enhance the ramie degumming efficiency of strain HG-49 for industrial application, this study systematically investigated its degumming process. The objectives were to elucidate the microbial degumming mechanisms and to pinpoint the key factors constraining efficiency improvement. The main research contents include: the growth curve of strain HG-49 during degumming; activity changes in pectinase, xylanase, mannanase, and cellulase; physicochemical property changes in the degumming solution (COD/reducing sugars, pH/dissolved oxygen, redox potential/conductivity); morphological and microstructural changes in ramie fibers (appearance, microscopic structure, FTIR, micro-FTIR, crystallinity, degree of polymerization, thermogravimetric analysis); and transcriptomic analysis of the degumming process. Based on these studies above, this study will put forward constructive strategies to improve the degumming efficiency of strain HG-49 for ramie bast processing.

## 2. Materials and Methods

### 2.1. Ramie Bast Fiber and Degumming Strain

The ramie bast fibers used in this study were derived from plants grown in Dazhou City, Sichuan Province, China. Prior to experimentation, all fiber samples were oven-dried at 60 °C to eliminate moisture. Degumming strain *P. carotovorum* HG-49, which was isolated by our research group, is maintained at CCTCC as M2017016.

### 2.2. Microbial Degumming System for Ramie Bast Fibers

Ramie bast (12 g) was added to 300 mL of distilled water and sterilized through autoclaving. After cooling, 15 mL of strain HG-49 cultured for 8 h was inoculated. Degumming was carried out at 30 °C and 180 rpm in a shaking incubator (Shanghai Zhichu Instrument Co., Ltd., Shanghai, China) for 16 h. Every 2 h, 5 mL of degumming liquor was withdrawn for the determination of strain biomass, enzyme activity, and physicochemical properties. All experiments were performed in triplicate.

Ramie bast (4 g) was added to 100 mL of distilled water in each of 27 flasks and sterilized through autoclaving. After cooling, 5 mL of strain HG-49 cultured for 8 h was inoculated into each flask. Degumming was conducted at 30 °C and 180 rpm for 16 h, and every 2 h, three flasks were taken out. The degummed ramie fibers were collected, washed, and dried for morphological and material property characterization.

### 2.3. Growth Curve Determination of Degumming Strain HG-49

The degumming liquor was withdrawn at 2 h intervals, the optical density at 600 nm (OD_600_) was determined using a Bio Tek microplate reader (Agilent, Santa Clara, CA, USA), and the growth curve was then plotted. The medium without strain HG-49 inoculation was set as the blank control.

### 2.4. Enzyme Activity

Pectinase, xylanase, mannase and cellulase activity were determined via the DNS assay by monitoring reducing sugar production from orange peel pectin, beechwood xylan, konjac powder, and sodium carboxymethyl cellulose (CMC), respectively. Enzyme activity (U) was defined as the release of 1.0 μg reducing sugars per minute [21]. The reaction mixture was 0.1 M phosphate buffer (pH 7.0) contained with 0.5% of pectin, xylan, mannan and CMC, respectively, incubated at 37 °C for 10 min. Each assay was run in triplicate.

### 2.5. Reducing Sugar and COD Determination

The reducing sugar content during the degumming process was determined according to the DNS method [22]. Chemical oxygen demand (COD) was determined using the potassium dichromate stock solution method. Samples of ramie degumming liquor were collected at 2 h intervals for the above measurements.

### 2.6. Determination of Physicochemical Properties

Dissolved oxygen (DO), pH, electrical conductivity (EC), and oxidation–reduction potential (ORP) of the degumming liquids were measured using a Hach multi-parameter meter (Hach, Loveland, CO, USA) at 2 h intervals.

### 2.7. Characteristic Analysis of Ramie Fibers

#### 2.7.1. Gum Composition Analysis

The variably treated ramie fiber samples were assayed for their total gum content as well as their individual pectin, hemicellulose, lignin, water-soluble, and wax components, strictly adhering to the China textile criteria (GB 5889-86) for quantitative evaluation [23].

#### 2.7.2. Microstructural Characterization Analysis of Ramie Fibers

For surface morphology characterization, the ramie fiber specimens were first gold-coated through ion sputtering, then subjected to a scanning electron microscope (SEM) (Nova NanoSEM 450, FEI Company, Hillsboro, NJ, USA) at an accelerating voltage of 2.0 kV and a magnification of 1000×, all following established SEM procedures [24].

#### 2.7.3. ATR FTIR Analysis

Attenuated total reflectance Fourier transform infrared (ATR-FTIR) spectroscopy (Nicolet iS50R, Thermo Scientific, Waltham, MA, USA) was employed to characterize the functional groups of ramie fibers, with spectra acquired over 500–4000 cm^−1^ at 4 cm^−1^ resolution [25,26]. The resulting data were further subjected to Gaussian peak fitting for hydrogen bond evaluation.

#### 2.7.4. X-Ray Diffraction (XRD) Analysis

X-ray diffraction (XRD) patterns of the samples were acquired on a powder diffractometer operating with Cu Kα radiation (40 kV, 40 mA), with data collected over the 2θ range of 5–40° at a scanning speed of 0.08°/s (Empyrean, PANalytical B.V, Almelo, Netherlands). Based on the obtained diffractograms, the cellulose crystallinity index was calculated from the peak intensity ratio using the Segal equation: CrI (%) = (Icr/(Icr + Iam)) × 100. Estimation of the average crystallite size was performed by analyzing the XRD diffractograms with the Scherrer equation [27].

#### 2.7.5. Thermal Stability Analysis

Thermogravimetric analysis (TGA) was performed on a Pyris1 instrument (PerkinElmer Instruments, Shanghai, China). The dried ramie samples were then subjected to thermal degradation by heating from 50 °C to 800 °C at a constant rate of 10 °C/min in a nitrogen purge (40 mL/min) [28].

#### 2.7.6. Fourier Transform Micro-Infrared Imaging (Micro-FTIR)

For micro-FTIR analysis, a single ramie fiber was first extracted and mounted onto the detector of a Nicolet iN10 spectrometer (Thermo Scientific, Waltham, MA, USA). Surface scanning was then carried out across a 5 × 18 measurement grid over the wavenumber range of 500–4000 cm^−1^ [27,28]. The acquired micro-region spectra were subsequently labeled and recorded using OMNIC Picta software 1.7 (ThermoFisher, Waltham, MA, USA).

#### 2.7.7. Degree of Polymerization (DP) Test of Ramie Fibers

DP testing was carried out in accordance with GB 5888-86 [29]. The procedure began with the dewaxing of the ramie fibers using a benzene–ethanol mixture (2:1, *v*/*v*), followed by size reduction to <1 mm and drying at 60 °C to constant weight. The pretreated fibers (40 mg) were then dissolved in 20 mL of 0.5 M BCH solution over 1 h. For viscosity measurement, 6.5 mL of the dissolved sample was introduced into an Ostwald viscometer (Huabo, Qingdao, China), and the efflux time was recorded, with the 0.5 M BCH solution used as the control [30].

### 2.8. Transcriptome Sequencing, Analysis and qPCR Validation

#### 2.8.1. RNA Extraction, Library Construction, and Transcriptome Sequencing

Cell samples of strain HG-49 were harvested at 4 h, 6 h, 8 h, 10 h, 12 h, 14 h, and 16 h during the degumming process, with three biological replicates per time point. Total RNA was extracted using the RNA Easy Fast kit (TianGen DP451, Beijing, China), its purity and concentration were determined using a NanoDrop 2000 spectrophotometer (ThermoFisher, Waltham, MA, USA), and RNA integrity was evaluated through agarose gel electrophoresis. Qualified RNA samples were sent to Novogene Company to construct a library based on standardized process. Paired-end sequencing was carried out on the Illumina HiSeq TM2500 (Illumina, San Diego, CA, USA).

#### 2.8.2. Data Processing and Analysis

Raw reads were filtered to remove adapter sequences and low-quality reads using Trimmomatic. Clean reads were aligned to the reference genome of strain HG-49 using Bowtie 2. Gene expression levels were quantified as transcripts per million (TPM) and fragments per kilobase of transcript per million mapped reads (FPKM) using StringTie 2.2.1. Gene Ontology (GO) and Kyoto Encyclopedia of Genes and Genomes (KEGG) enrichment analyses were performed using clusterProfiler. All statistical analyses were conducted in R 4.0.0 [31,32].

#### 2.8.3. Quantitative PCR Validation

Degumming enzyme genes (Table S1) with obvious transcriptional trends were selected from the transcriptome data. Primers were designed using Primer Premier 5 according to the corresponding gene sequences (Table S2). Quantitative PCR (qPCR) was performed on RNA samples collected from 4 to 16 h of ramie bast fibers’ degumming using an Applied Biosystems StepOne Real-Time PCR System (ABI, Waltham, MA, USA), with three replicates per sample. The data were analyzed using StepOne Software v2.3.

## 3. Results and Discussion

### 3.1. Dynamic Changes Analysis of Degumming Process

The microbial degumming of ramie bast fibers is an interactive process involving microorganisms, ramie bast fibers, and the degumming system. Factors such as microbial concentration, changes in degumming enzyme activity, the degradation of various gum substances, as well as variations in physicochemical properties of the degumming system, including dissolved oxygen, pH, conductivity, and oxidation–reduction potential, significantly influence the degumming effect. Therefore, systematically monitoring these indicators during the degumming process is crucial for revealing the dynamic rules of microbial degumming.

#### 3.1.1. The Microbial Growth Analysis

**Figure 1:**
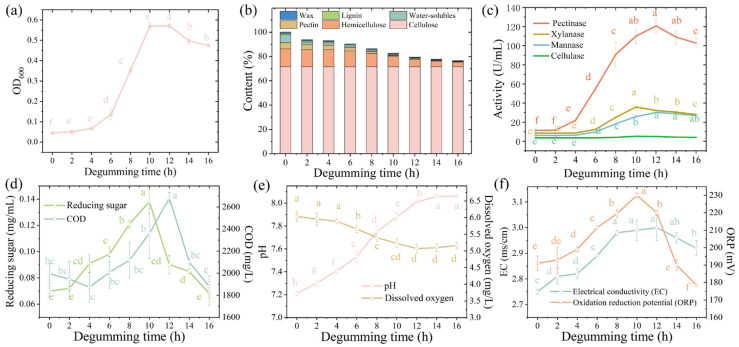
Dynamic change analysis of ramie bast fiber’s degumming process by *Pectobacterium carotovorum* HG-49. (**a**) Growth curve of strain HG-49; (**b**) gum content changes; (**c**) activities of pectinase, mannase, xylanase and cellulase; (**d**) reducing sugar contents and COD; (**e**) pH and dissolved oxygen; (**f**) electrical conductivity and oxidation–reduction potential. (a–h: Significant differences analysis among multiple samples based on Statistical Product and Service Solutions (SPSS) version 21 one-factor ANOVA, *p* < 0.05).

To understand the growth dynamics of strain HG-49 and the degradation of gum substances during the degumming process, the biomass of the strain and the total gum degradation rate were measured every 2 h. In the first 4 h of degumming, the biomass of the strain showed little change, indicating that HG-49 was adapting to the environment in the lag phase. From 6 to 10 h, HG-49 entered the logarithmic growth phase, with rapid proliferation of bacterial cells. By 12 h, the biomass reached its peak. Subsequently, the biomass gradually declined but remained at a high level. By 16 h, the biomass declined to minimum value (Figure 1a).

#### 3.1.2. The Gum Removal Ratio Change Analysis

To understand the degradation patterns of different gum components in ramie bast and identify the specific recalcitrant residues, the contents of pectin, hemicellulose, water-soluble substances, lignin, and wax were measured every 2 h during degumming. Pectin content began to decrease at 4 h, with rapid degradation occurring between 4 and 12 h. By 16 h, the removal rate of pectin reached 97.05%. Water-soluble substances also showed a significant decline starting at 4 h, with the fastest degradation between 4 and 8 h, achieving a 98.45% removal rate by 16 h. Hemicellulose content remained largely unchanged in the first 4 h but started decreasing at 6 h, with accelerated degradation between 6 and 14 h, reaching a 73.54% removal rate at 16 h. Both lignin and wax began degrading at 6 h, with their fastest removal occurring between 8 and 14 h, and their final contents dropping below 0.5% by 16 h (Figure 1b). These results demonstrate that pectin and water-soluble substances were almost completely removed, while the remaining gum primarily consisted of hemicellulose.

#### 3.1.3. The Degumming Enzyme Activity Change Analysis

The essence of microbial degumming lies in the degradation of gum substances by degumming enzymes. The activities of pectinase, mannanase, xylanase, and cellulase in the degumming solution were measured every 2 h. Pectinase activity began to rise at 4 h, with the fastest increase occurring between 6 and 10 h, reaching a peak of 120.75 U/mL at 12 h. Afterward, the enzyme activity slightly declined but remained at a relatively high level (Figure 1c). In comparison, mannanase and xylanase exhibited delayed expression and significantly lower activity than pectinase (Figure 1c). Their activity only showed a noticeable increase starting from 6 h, with the logarithmic phase of activity rise occurring between 6 and 10 h. They reached their peak values of 35.85 U/mL and 30.20 U/mL at 10 and 12 h, respectively, followed by a continuous decline. Cellulase began to be expressed at 6 h but remained at a very low level, around 5 U/mL (Figure 1c). The enzyme activity results indicate that strain HG-49 exhibits high pectinase activity during the degumming process, while mannanase and xylanase activities are relatively low, only about one-third of pectinase activity. Cellulase activity remained extremely low throughout, ensuring no damage to the ramie fibers. The enzyme activity curves align with the gum degradation curves: the high pectinase activity led to thorough pectin removal, whereas the low mannanase and xylanase activities resulted in residual hemicellulose that was difficult to completely eliminate. These results demonstrate that the key to the efficient degumming capability of strain HG-49 lies in its high pectinase activity. At the same time, the low hemicellulase activity is a critical factor limiting its degumming efficiency.

#### 3.1.4. Physical and Chemical Property Change Analysis

The degradation of gummy substances during degumming generates reducing sugars, the concentration of which can serve as an indicator of the extent of gum degradation. The results showed that reducing sugars began to accumulate at 4 h of degumming (0.091 mg/L), reached a peak at 10 h (0.138 mg/L), and then decreased sharply, becoming barely detectable by 16 h (Figure 1d). This trend indicates that gum degradation commenced at 4 h, leading to sugar accumulation; however, after 12 h, the rate of sugar consumption by the strain exceeded the rate of gum degradation, resulting in a rapid decline in sugar concentration. The degumming broth following 10 h of treatment with strain HG-49 was used to analyze the monosaccharide composition through HPLC, and the results showed that the ramie degumming liquid comprised mannose, rhamnose, glucuronic acid, glucose, galactose, xylose, and arabinose (Figure S1). The chemical oxygen demand (COD) level exhibited a slight initial decrease, followed by an increase and then a sharp decline (Figure 1d), a trend that was generally consistent with that of the reducing sugar concentration.

The pH and dissolved oxygen (DO) levels of the degumming system are critical physicochemical factors affecting bacterial growth. To understand the changes in pH and DO during the degumming process by strain HG-49, these parameters were measured every 2 h. The initial pH of the degumming solution was 7.21. As degumming progressed, the pH steadily increased, peaking at 8.06 after 14 h, indicating a slightly alkaline environment, before slightly decreasing until the end of the process (Figure 1e). This pH rise may be attributed to the release of alkaline substances, such as OH^−^, resulting from the depolymerization of gum polysaccharides. Notably, the period from 4 to 12 h marked the logarithmic phase of pH increase, coinciding with the primary stages of bacterial growth and gum degradation. According to reports, *Pectobacterium carotovorum* (the strain used) grows optimally at neutral pH but can tolerate a range of 5.3–9.2. Thus, the observed pH variation (7.21–8.06) had minimal impact on bacterial growth. The initial DO was 6.03 mg/L, remaining stable for the first 4 h before declining between 6 and 12 h, reaching a minimum of 5.07 mg/L at 12 h, followed by a slight rebound. Since bacterial growth consumes significant oxygen, the DO decrease from 6 h onward aligns with strain HG-49 entering its logarithmic growth phase at 6 h and peaking in biomass at 12 h. However, continuous aeration ensured DO levels remained above 5 mg/L, providing sufficient oxygen for bacterial proliferation. These results indicate that the pH and DO fluctuations in the degumming system had little adverse effect on strain’s growth.

Conductivity is commonly used to indirectly estimate the total concentration of ionic components in aqueous solutions, where within a certain range, higher ion concentrations correspond to greater conductivity. Redox potential reflects the macroscopic oxidation–reduction properties of all substances in a solution, with higher redox potentials indicating stronger oxidizing capacities. The degradation of ramie gum polysaccharides is itself a redox reaction that affects the redox potential of the degumming system, while simultaneously releasing various ions into the degumming solution that influence its conductivity. The results revealed an initial redox potential of 191 mV, which remained nearly unchanged during the first 4 h, then increased sharply from 4 h to 10 h, reaching a peak of 230 mV at 10 h, followed by a rapid decline starting at 12 h and dropping to 178 mV by 16 h (Figure 1f). As the period from 4 to 12 h corresponded to active bacterial growth, high expression of degumming enzymes, and rapid gum degradation, all of which involved redox reactions that drive the sharp increase in redox potential. After 12 h, the strain entered the decline phase, with reductions in biomass, enzyme activity, and gum degradation rates, consequently leading to the observed decrease in redox potential.

The initial conductivity of the degumming system was 2.75 ms/cm, and began to increase after 2 h due to the dissolution of water-solubles from ramie bast into the degumming solution. The conductivity showed no significant change at 2–4 h because strain HG-49 was still in the lag phase with low enzyme activity, resulting in minimal gum degradation and no substantial ion release into the solution. From 4 to 12 h, the conductivity steadily increased, coinciding with the period of rapid bacterial growth and intensive gum degradation. During this phase, various degradation products were released into the degumming solution, contributing to the rise in conductivity. After 12 h, the conductivity began to decline, corresponding to reduced bacterial biomass, enzyme activity, and gum degradation rates, which aligned with the findings presented above (Figure 1f).

### 3.2. Material Characterization Analysis of Ramie Fibers in Degumming Process

The changes in ramie fibers during the degumming process served as crucial indicators for evaluating degumming effects and elucidating degumming mechanisms. In this study, comprehensive and systematic analyses toward the transformations of ramie fibers were conducted through morphological observation, surface functional group determination, hydrogen bond analysis, micro-infrared spectroscopy, crystallinity and polymerization degree assessment, as well as thermodynamic property characterization.

#### 3.2.1. Morphological Analysis of Ramie Fibers

**Figure 2:**
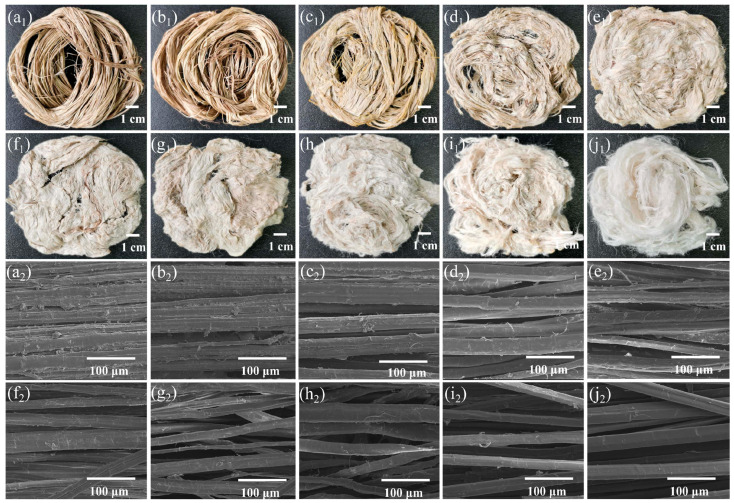
Morphological photographs and scanning electron microscope photos (400×) of ramie fibers degummed by *Pectobacterium carotovorum* HG-49. (**a_1_**,**a_2_**) Ramie bast fibers, RBF; (**b_1_**,**b_2_**) ramie fibers degummed for 2 h; (**c_1_**,**c_2_**) ramie fibers degummed for 4 h; (**d_1_**,**d_2_**) ramie fibers degummed for 6 h; (**e_1_**,**e_2_**) ramie fibers degummed for 8 h; (**f_1_**,**f_2_**) ramie fibers degummed for 10 h; (**g_1_**,**g_2_**) ramie fibers degummed for 12 h; (**h_1_**,**h_2_**) ramie fibers degummed for 14 h; (**i_1_**,**i_2_**) ramie fibers degummed for 16 h; (**j_1_**,**j_2_**) refined ramie fibers.

Ramie fibers were sampled at 2 h intervals during the degumming process, washed with clean water, dried and photographed. Observation of fiber morphology changes revealed that the fibers showed little difference from the raw ramie during the first 2 h of degumming. As degumming progressed, significant morphological changes became apparent starting from 6 h, with the yellowish-brown gum gradually diminishing, the fibers beginning to separate, and their color progressively whitening. Compared with refined ramie fibers, the fibers degummed for 16 h retained only small amounts of yellowish-brown gum, and their whiteness and dispersion degree were already close to those (Figure 2).

To gain clearer insight into the morphological changes in ramie fibers during the degumming process, scanning electron microscopy (SEM) was employed for 400× magnification observation. The SEM images clearly revealed that raw ramie fibers were bound together by a thick gum layer on their surface, making individual fibers nearly indistinguishable. Little change was observed in the fibers during the first 4 h of degumming. Starting from 6 h, significant fiber separation became apparent, with the gum coating on fiber surfaces progressively diminishing. After 16 h of degumming, only small amounts of residual gum remained attached to the fiber surfaces (Figure 2). Based on the gum degradation curve results, these residual substances were primarily identified as hemicellulose. In contrast, the refined ramie fibers obtained through subsequent alkali boiling, bleaching, and oiling treatment exhibited completely clean and smooth surfaces with no visible gum residues. The SEM results further confirmed the excellent degumming capability of strain HG-49, demonstrating that the period from 6 to 12 h were the main phase of gum degradation, which aligned well with the findings above.

#### 3.2.2. Surface Functional Groups Analysis of Ramie Fibers

ATR-FTIR was employed to conduct infrared spectral absorption analysis on ramie fibers at different degumming stages and refined ramie samples. The presence and variations in functional groups and chemical bonds in ramie fibers were qualitatively and quantitatively characterized based on absorption peak intensities at different wavelengths. According to literature reports [26,33], the OH absorption peak near 3300 cm^−1^ primarily originates from cellulose and hemicellulose; the CH peak around 2850 cm^−1^ mainly represents lipid waxes; the carboxylate peak at 1640 cm^−1^ is predominantly found in pectin and hemicellulose; aromatic ring vibrations (1400–1550 cm^−1^) indicate lignin content; and pyranose ring absorptions (1000–1200 cm^−1^) are characteristic of hexoses like glucose, mannose, and galactose that mainly represent cellulose and hemicellulose structures.

**Figure 3:**
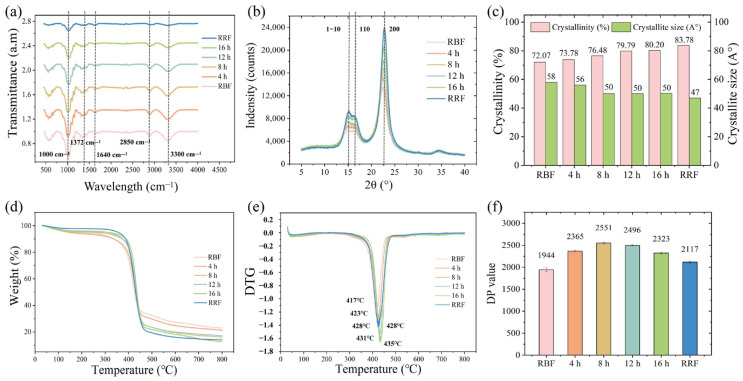
Characteristic analysis of ramie fibers degummed by *Pectobacterium carotovorum* HG-49. (**a**) Attenuated total reflectance Fourier transform infrared spectroscopy (ATR FTIR); (**b**) X-ray diffraction (XRD); (**c**) crystallinity and crystallinity size; (**d**) thermo-gravimetric analysis (TGA); (**e**) differential thermogravimetric analysis (DTG); (**f**) degree of polymerization (DP). (RBF: ramie bast fibers; 4–16 h: ramie fibers degummed for 4–16 h; RRF: refined ramie fibers; dash line: characteristic absorption peak).

All monitored absorption peaks progressively decreased during degumming, demonstrating gradual degradation of gummy components including pectin, hemicellulose, lignin, and lipid waxes. At 4 h of degumming, peak intensities showed negligible changes compared to initial values. By 8 h, significant reductions occurred across all wavelengths, with further decreases observed at 16 h. Compared to refined ramie, the 16 h degummed fibers retained relatively strong absorptions only at 3300 cm^−1^ and 1000 cm^−1^ (representing cellulose and hemicellulose), while other peaks closely matched those of refined ramie (Figure 3a). These findings confirmed that HG-49 strain treatment for 16 h effectively removed most gummy substances from ramie bast, with residual components being primarily hemicellulose.

#### 3.2.3. Crystallinity Analysis of Ramie Fibers

To determine whether strain HG-49s degumming process damages ramie cellulose, X-ray diffraction (XRD) was employed to analyze ramie fiber samples at different degumming stages and refined ramie, characterizing the integrity and crystallinity of ramie cellulose. The X-ray diffraction profiles displayed three characteristic reflections at 14.8°, 16.8°, and 22.8°, which were assigned to the (1–10), (110), and (200) crystallographic planes of cellulose I, respectively. A comparison of the six diffraction patterns revealed no discernible shifts or emergence of new peaks, confirming that the crystalline structure and cellulose allomorph of the ramie fibers were preserved throughout the degumming process (Figure 3b). According to reports [28,34], this peak specifically corresponds to crystalline cellulose, confirming that strain HG-49s degumming does not compromise ramie cellulose.

Crystallinity and size calculations revealed that raw ramie had value of 72.07% and 58, while fibers degummed for 4 h, 8 h, 12 h, 16 h, and refined ramie showed crystallinity and size values of 73.78%/56, 76.48%/50, 79.79%/50, 80.20%/50, and 83.78%/50, respectively (Figure 3c). Crystallinity progressively increased during degumming: the 4 h sample showed minimal improvement over raw ramie, whereas 8 h and 12 h samples exhibited significant jumps. The 16 h sample’s crystallinity improved only marginally compared to 12 h. Crystallinity size exhibited a gradually decreasing trend, with values becoming progressively smaller as the degumming process proceeded, and reaching the minimum in the refined ramie fibers. These results align with the gum removal rates in the preceding section, confirming that 6–12 h marks the primary phase of gum degradation and cellulose crystallinity enhancement.

The findings demonstrated that as pectin, hemicellulose, lignin, and other gummy substances progressively removed, ramie cellulose purity improved, its structural arrangement tightened, crystallinity rose, and crystallinity size lessened. However, the Segal method used for crystallinity determination has inherent limitations, because this empirical approach calculates crystallinity index solely from the ratio of the crystalline peak intensity to the amorphous background, without accounting for paracrystalline regions or different cellulose polymorphs.

#### 3.2.4. Thermal Stability Analysis of Ramie Fibers

The above crystallinity results demonstrate that the cellulosic integrity of ramie fibers in degumming process is well retained. Combined with SEM and FTIR results, the structure of ramie fibers are considered to be intact without damages. Therefore, a thermal stability analysis of the ramie fibers was conducted. TGA is widely employed as a reliable method to evaluate the thermal stability of various substances [29,35]. Results of TGA (Figure 3d) and DTG (Figure 3e) revealed that the ramie bast fibers had the lowest degradation temperature of 417 °C. The degradation temperatures of ramie fibers after 4, 8, 12, and 16 h of degumming were 423, 428, 431, and 435 °C, respectively, with the highest value observed at 16 h. The degradation temperature of refined ramie fiber exhibited a slight decrease. Throughout the microbial degumming process, the degradation temperature of the ramie fibers showed an overall upward trend, which can be attributed to the progressive removal of non-fibrous components (such as water-solubles, pectin, and hemicellulose) and the consequent purification of the cellulose, as well as the increased crystallinity and strengthened intramolecular hydrogen bonding of the cellulose. The slight decline in degradation temperature of the refined ramie fibers was likely due to fiber damage incurred during the subsequent alkali boiling and bleaching treatments.

#### 3.2.5. Degree of Polymerization of Ramie Fibers

Degree of polymerization (DP) represents the mean polymerization of the total fiber mass, including non-cellulosic fractions. A reduction in non-cellulosic content (pectin, solubles, and hemicellulose) is generally associated with an increase in DP [36,37]. As shown by the results, ramie bast fiber exhibited the minimum DP value of 1944. After degumming for 4, 8, 12, and 16 h, the DP values of the ramie fibers were 2365, 2551, 2496, and 2323, respectively, while the refined ramie fibers had a DP value of 2117 (Figure 3f). The DP value reached its highest point at 8 h, exhibiting an overall trend of first increasing and then decreasing. The initial increase in DP value can be attributed to the extensive removal of low-polymerization gummy substances, such as water-solubles and pectin. The slight decline observed in the later stage is likely due to the degradation of high-polymerization components, including lignin, hemicellulose, and even a portion of the cellulose itself, ultimately yielding relatively pure cellulose.

#### 3.2.6. Hydrogen Bond Analysis of Ramie Fibers

**Figure 4:**
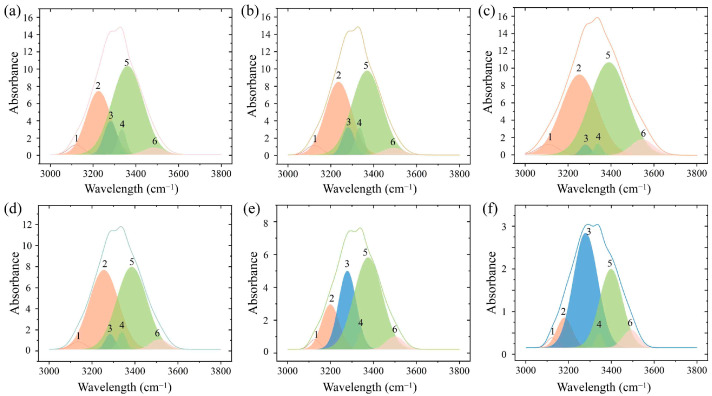
Hydrogen bond analysis of ramie fibers degummed by *Pectobacterium carotovorum* HG-49. (**a**) Ramie bast fibers, RBF; (**b**) ramie fibers degummed for 4 h; (**c**) ramie fibers degummed for 8 h; (**d**) ramie fibers degummed for 12 h; (**e**) ramie fibers degummed for 16 h; (**f**) refined ramie fibers. (1, 5: intermolecular hydrogen bond; 2, 3, 4: intramolecular hydrogen bond; 6: free OH groups).

Lignocellulosic materials are characterized by abundant intramolecular and intermolecular hydrogen bonds, as well as free hydroxyl groups. To some extent, variations in hydrogen bond content can reflect changes in the major components, including cellulose, hemicellulose, lignin, and pectin [38]. According to the spectral assignments reports [39], intramolecular hydrogen bonds are typically observed in the 3100–3550 cm^−1^ region, intermolecular hydrogen bonds near 3300 cm^−1^, and free hydroxyl groups around 3600 cm^−1^. The Gaussian deconvolution of the 3000–3800 cm-1 spectral region was carried out to quantitatively assess the hydrogen bond distribution in ramie fibers at various degumming stages (Figure 4). A consistent decline was observed in the peak area of free hydroxyls (peak 6) with increasing degumming time, which is attributable to the progressive elimination of non-cellulosic gums, including pectin, hemicellulose, and lignin. Likewise, the peak areas corresponding to intermolecular hydrogen bonds (peaks 1 and 5) decreased steadily, suggesting the successful breakdown and removal of lignin–polysaccharide matrices. In contrast, intramolecular hydrogen bonds, which are formed through cellulose–cellulose associations mediated by β-1,4 glycosidic linkages and are crucial for the structural integrity of cellulose polymers [40], displayed a gradual increase in relative proportion (peaks 2, 3, and 4; Table S3). This trend corroborated the effective removal of gummy impurities and the consequent purification of the cellulosic fraction.

#### 3.2.7. Micro-FTIR Analysis of Ramie Fibers

**Figure 5:**
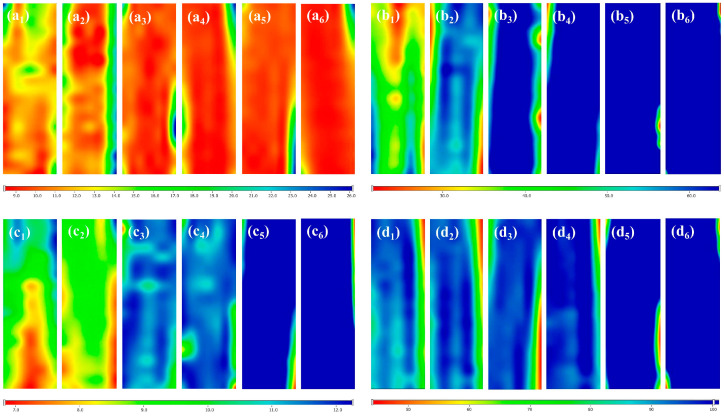
Fourier transform micro-infrared (Micro-FTIR) analysis of ramie fibers degummed by *Pectobacterium carotovorum* HG-49. (**a**) Cellulose; (**b**) pectin; (**c**) hemicellulose; (**d**) lignin. (**1**: ramie bast fibers, RBF; **2**: ramie fibers degummed for 4 h; **3**: ramie fibers degummed for 8 h; **4**: ramie fibers degummed for 12 h; **5**: ramie fibers degummed for 16 h; **6**: refined ramie fibers).

Micro-FTIR mapping enables visual characterization of the distribution and relative abundance of various chemical components across the ramie fiber surface. In the resulting color-coded maps, red indicates the highest absorbance intensity, followed by yellow, green, and blue, which represents the lowest. In essence, regions with higher component concentrations appear redder, whereas those with lower concentrations appear bluer. In line with well-established spectroscopic assignments, the absorption bands located at 1732, 1640, 1506, and 1372 cm^−1^ are indicative of hemicellulosic xylan, pectin, the aromatic skeleton characteristic of lignin, and the C–O–C linkages within cellulose, respectively [41,42]. As degumming proceeded, the micro-FTIR maps of cellulose (Figure 5a) in ramie fibers became progressively redder after 4, 8, 12, and 16 h of treatment, and the average absorbance increased from 0.521 to 0.614, the refined ramie fiber exhibiting the reddest map with average absorbance of 0.652. This color shift indicated the continuous removal of gummy components and the progressive purification of cellulose. The micro-FTIR maps representing pectin (Figure 5b), hemicellulose (Figure 5c), and lignin (Figure 5d) exhibited progressively bluer coloration and the average absorbance decreased to 0.009, 0.045 and 0.016 after degumming 16 h, respectively (Table S4). Notably, the pectin map became almost entirely blue after 16 h of degumming, indicating thorough removal. In contrast, the maps for hemicellulose and lignin still retained small amounts of red, yellow, and green even after 16 h, suggesting incomplete removal with partial residues remaining. These results are in accordance with that of ATR FTIR and chemical composition analysis.

#### 3.2.8. Textile Parameters of Ramie Fibers

The quality of ramie fibers is primarily governed by three textile performance indicators: bundle breaking tenacity, whiteness, and residual gum content. The first-class grade, as defined by Chinese textile criteria, stipulates that these parameters must meet the following standards: residual gum content ≤ 2%, breaking tenacity ≥ 4.5 cN/dtex, and whiteness ≥50. The bio-degummed refined ramie fibers exhibited a bundle breaking tenacity of 4.95 ± 0.11 cN/dtex, a whiteness of 57.5 ± 1.1, and a residual gum content of 1.98 ± 0.14%, all of which meet the first-class level of the Chinese textile criteria, demonstrating that degumming with strain HG-49 imposes negligible damage on the ramie fibers.

In conclusion, this above study systematically investigated strain HG-49s degumming process, including dynamic changes analysis of degumming process (growth curve, gum degradation curve, enzyme activities, physicochemical parameters); material characterization analysis of ramie fibers in degumming process (SEM, FTIR, Micro-FTIR, XRD, TGA, DP), all of which were critical factors influencing the degumming efficiency of strain HG-49 during ramie fibers’ degumming. Subsequent correlation analysis revealed that pectinase activity, xylanase activity, and cellulose crystallinity were the most strongly associated with degumming efficiency, yielding correlation coefficients of 0.92, 0.95, and 0.98, respectively (Figure S5).

### 3.3. Transcriptome Analysis of Degumming Process by Strain HG-49

Our previous study has confirmed the types and quantities of degumming enzymes possessed by strain HG-49. However, the expression levels and temporal dynamics of these enzymes during the degumming process, the degradation and metabolic pathways of ramie gums remained unclear. Given that strain HG-49s growth, enzyme expression, and gum degradation commenced at 4 h of degumming, transcriptome sequencing and analysis on samples spanning 4 h to 16 h of the degumming process were conducted to elucidate the regulatory patterns of microbial degumming at the transcriptional level.

#### 3.3.1. Overview of Transcriptome in Degumming Process

To monitor transcriptomic changes during degumming, bacterial samples of strain HG-49 were collected at 2 h intervals from 4 to 16 h, and total RNA was extracted from each. Agarose gel electrophoresis showed clear 23S and 16S rRNA bands for all 21 samples, without smearing, trailing, or additional bands, confirming RNA integrity (Figure S2). Absorbance measurements using a NanoDrop 2000 revealed OD260/OD280 and OD260/OD230 ratios within acceptable ranges, indicating that the RNA preparations were free from protein, organic, and salt contamination (Tables S5 and S6). Overall, the total RNA obtained from strain HG-49 was of high integrity, purity, and concentration, satisfying the criteria for transcriptome sequencing. Sequencing was carried out on the Illumina HiSeq 2500 platform, generating 235,750,588 raw reads, which were reduced to 229,613,594 clean reads after quality trimming and filtering. The GC content was approximately 50%, reflecting robust sequencing quality. The clean reads were mapped to the reference genome using Bowtie 2, achieving mapping rates above 98% for all 21 samples, thus enabling reliable downstream analyses (Table S7).

**Figure 6:**
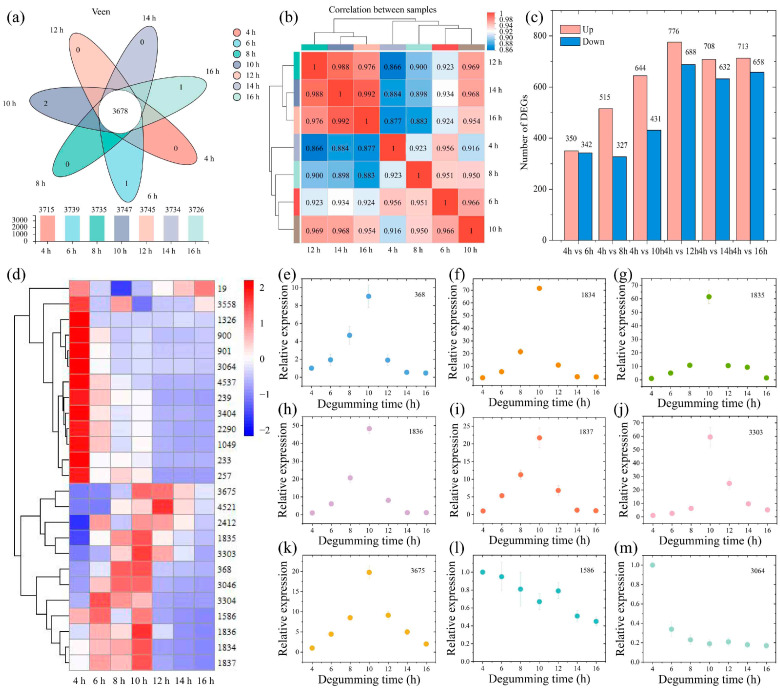
Transcriptome analysis of degumming process by *Pectobacterium carotovorum* HG-49. (**a**) Venn diagram; (**b**) correlation between samples; (**c**) differential expression genes (DEGs) analysis; (**d**) heatmap analysis of genes expression for degumming enzymes; (**e**–**m**) quantitative real-time PCR analysis of degumming enzyme gene expression. (19-4537: gene IDs of degumming enzymes in strain HG-49).

Venn diagram analysis of the seven time points (4–16 h) revealed that the number of expressed genes ranged from 3715 to 3747, with a peak at 10 h (3747 genes) followed by a gradual decline (Figure 6a). Intersample variation was minimal, with 3678 genes shared across all time points and only a negligible number of uniquely expressed genes. Correlation analysis indicated that the transcriptomic data exhibited good reproducibility across all samples (Figure 6b). Differential expression analysis, using the 4 h sample as the baseline, showed that the number of differential expression genes (DEGs) increased steadily from 6 to 12 h, with upregulated genes totaling 350, 515, 644, and 766, respectively (Figure 6c). The maximum number of DEGs occurred at 12 h, after which the count decreased, a trend that aligns well with the overall degumming dynamics of strain HG-49.

#### 3.3.2. Transcriptional Changes in Degumming Enzymes

A heatmap of degumming enzyme gene expression during the degumming process was constructed based on transcriptomic data. Seven pectate lyase genes, designated 368, 1834, 1835, 1836, 1837, 3303, and 3675, exhibited FPKM values exceeding 1000 (Figure 6d). Their transcript levels rose progressively from 4 h, reached a maximum at 10 h, and then decreased thereafter. qPCR validation using the 2^−∆∆CT^ method confirmed these expression trends, a pattern that aligned well with the dynamics of pectinase activity and pectin removal rate, thereby implicating them as candidate key degumming enzymes. Among these genes, 1834, 1835, 1836, and 3303 displayed markedly higher relative expression than the others, with 10 h transcript levels representing 71.5-, 61.5-, 48.3-, and 59.4-fold increases over the 4 h point, respectively (Figure 6e–k). In parallel, two β-xylosidase genes (1586 and 3064) were also assessed through qPCR (Figure 6l,m). In contrast to pectate lyase genes, both β-xylosidase genes exhibited down-regulated expression from 6 to 16 h relative to the 4 h control, with transcript levels progressively declining throughout degumming, a trend that deviated from the xylanase activity. Therefore, based on the combined evidence from enzymatic activity assays, transcriptomic analysis, and qPCR validation, the seven pectate lyase genes (368, 1834, 1835, 1836, 1837, 3303, and 3675) were identified as the principal degumming enzymes, while the two β-xylosidase genes (1586 and 3064) were not found to play a critical role. Accordingly, strain HG-49 may possess multifunctional enzymes with xylanase activity, as activities of xylanase reached 35.85U/mL at degumming time 10 h. In addition, numerous such multifunctional enzymes have been documented among glycoside hydrolases, and this inference is further supported by our previous finding that an endoglucanase (Gene id. 4349) exhibits both mannanase and xylanase activities [43].

#### 3.3.3. The Microbial Degumming Mechanism for Ramie Bast Fibers

**Figure 7:**
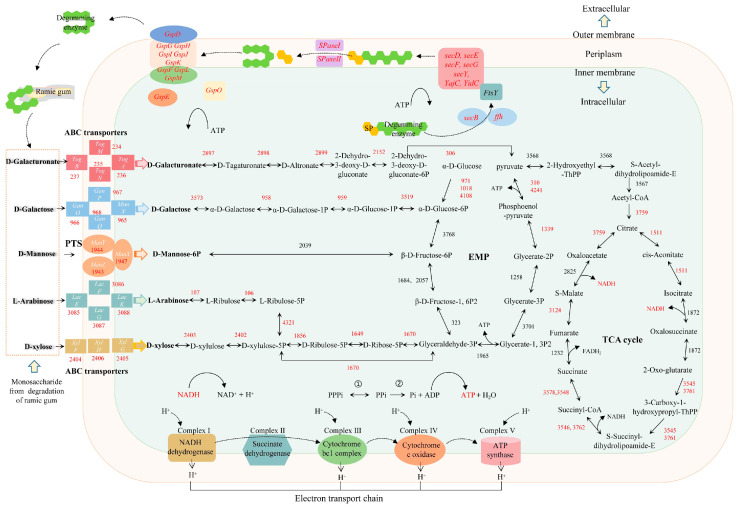
Schematic diagram of the degumming mechanism for *Pectobacterium carotovorum* HG-49. (106-4321: gene IDs from genome of strain HG-49 with Genbank accession number of CP032619; red marker: upregulated expression genes at 4 h vs. 12 h; black marker: genes expression level has not changed; ABC transporters: ATP-binding cassette transporters; PTS: phosphotransferase system; EMP: embden meyerhof parnas; TCA: tricarboxylic acid).

Transcriptomic analysis revealed that strain HG-49 possesses a complete set of genes encoding the type II secretion system, all of which were almost uniformly upregulated when comparing the 4 h and 12 h degumming time points. Therefore, it is speculated that the degumming enzymes are secreted extracellularly via the type II secretion system, where they subsequently degrade the gums in ramie bast. The resulting small-molecule monosaccharides, including galacturonic acid, galactose, mannose, xylose, and arabinose (HPLC analysis confirmed the presence of these monosaccharides in the degumming liquor, Figure S1) are subsequently transported into strain HG-49s cell through a complete transmembrane transport machinery, primarily involving the ATP-binding cassette (ABC) transporter pathway and the phosphotransferase system (PTS). Once internalized, these monosaccharides undergo sequential catabolism via embden meyerhof parnas (EMP) and the tricarboxylic acid (TCA) cycle. The reducing equivalents (NADH) generated during this process drive oxidative phosphorylation, thereby converting ADP to ATP to sustain cellular energy demands. During the degumming process, a majority of genes associated with secreted protein production, monosaccharide transmembrane transport and catabolism, as well as those encoding the electron transport chain core enzymes, including NADH dehydrogenase, cytochrome C oxidase, and ATP synthase, showed upregulated expression patterns (Figure 7).

Collectively, these findings elucidate the molecular mechanism underlying the microbial degumming paradigm, conceptualized as “bacteria produce enzymes, enzymes degrade gum, and gum nourishes bacteria”.

### 3.4. The Proposal of High-Efficiency Degumming Strategy for Strain HG-49

Biological degumming represents the future development trend of ramie fibers’ industrial production [44]. Through systematic investigation of the degumming process, the key factors constraining the degumming efficiency of strain HG-49 were identified and summarized as follows. First, the strain exhibited a prolonged lag phase during the initial cultivation period, which delayed the onset of active degumming and extended the overall processing time. Second, the maximum biomass accumulation and the corresponding degumming enzyme activities remained relative low level, resulting in insufficient enzymatic attack on the gum components. Third, the hemicellulase system of strain HG-49 is incomplete, lacking certain key enzymes necessary for the complete degradation of hemicellulose, which restricted the overall gum removal rate. Fourth, the ramie bast fibers possess a dense and compact cell wall architecture, which acted as a physical barrier that limited the accessibility of degumming enzymes to their substrates and thereby impeded efficient enzymatic hydrolysis.

To address these limitations and further enhance the degumming performance of strain HG-49, the following strategies are proposed in this study. (1) Supplementation of the degumming system with pectin-rich nutrients is recommended to stimulate rapid proliferation of strain HG-49 during the early growth phase, thereby shortening the lag period and accelerating the onset of gum degradation. (2) Heterologous supplementation or genetic engineering of the strain’s hemicellulase system is suggested to compensate for the missing enzymatic components, enabling more complete cleavage of hemicellulosic bonds. (3) Establishment of an effective pretreatment method for ramie bast fibers is proposed to physically disrupt the compact cell wall structure, increase the surface area accessible to enzymes, and facilitate subsequent enzymatic hydrolysis.

Overall, the findings and proposed strategies presented in this work provide a theoretical framework for enhancing microbial degumming efficiency, thereby facilitating the transition of this environmentally friendly technology from laboratory research to practical industrial deployment.

## 4. Conclusions

In this study, the degumming of ramie bast fibers by *Pectobacterium carotovorum* HG-49 was systematically investigated, with the aim of elucidating the mechanism of the degumming process. Strain HG-49 exhibited a lag phase of 0–4 h and a logarithmic phase of 6–10 h, reaching peak biomass at 12 h. Pectin (97.05%) and water-soluble substances (98.45%) were nearly completely removed, whereas hemicellulose removal reached only 73.54%, rendering it the predominant component of the residual gum. Pectinase activity peaked at 120.75 U/mL, while mannanase (35.85 U/mL) and xylanase (30.20 U/mL) attained approximately one-quarter of that level; cellulase activity remained consistently negligible throughout the process. Scanning electron microscopy (SEM) revealed that the period from 6 to 12 h constituted the primary phase of gum degradation. Fourier-transform infrared spectroscopy (FTIR) and micro-FTIR analyses showed progressive decreases in the absorption peaks corresponding to pectin, hemicellulose, and lignin as degumming advanced. X-ray diffraction (XRD) analysis indicated that the crystallinity of ramie fibers increased from 72.07% to 80.02% with degumming progression. Thermogravimetric analysis (TGA) further demonstrated that the degradation temperature of the fibers rose from 417 °C to 435 °C. Collectively, these data confirm that as degumming proceeds, gummy substances, including pectin, hemicellulose, and lignin, are progressively eliminated, accompanied by a continuous enhancement in cellulose purity. Transcriptomic profiling additionally revealed that the low abundance and reduced expression levels of hemicellulases produced by strain HG-49 significantly constrained degumming performance. The primary factors constraining degumming efficiency were identified as: (i) an extended lag phase of strain HG-49, resulting in suboptimal biomass accumulation and insufficient enzymatic activity; (ii) an incomplete hemicellulolytic enzyme system; and (iii) the compact lignocellulosic architecture of ramie bast, which impedes effective enzyme penetration and action. To address these limitations, the following enhancement strategies are proposed: (1) supplementation with pectin-enriched substrates to accelerate bacterial proliferation and enzyme production; (2) engineering or supplementation of the hemicellulase system to achieve a more complete enzymatic repertoire; and (3) implementation of effective pretreatment methods to disrupt cell wall integrity and facilitate enzymatic hydrolysis. Collectively, these findings provide a theoretical foundation for optimizing microbial degumming performance and advancing its viability in industrial-scale applications.

## Data Availability

The original contributions presented in this study are included in the article/Supplementary Material. Further inquiries can be directed to the corresponding authors.

## Supplementary Materials

The following supporting information can be downloaded at: https://www.mdpi.com/article/10.3390/polym18141775/s1, Figure S1. The monosaccharide composition analysis through HPLC detecting. (a) Standard substance; (b) degumming liquids at 10 h. Figure S2. Agarose gel electrophoresis analysis of total RNA extracted from strain HG-49 at different time points of the degumming process; Figure S3. Comparison of growth curves of *Pectobacterium carotovorum* HG-49 in LB medium and during ramie degumming process. Figure S4. Transcriptomic KEGG pathway analysis comparing *Pectobacterium carotovorum* HG-49 at 4 h versus 12 h of ramie degumming. (Genes in red boxes: upregulated expression, genes in green boxes: downregulated expression, genes in black boxes: absent from strain HG-49). Figure S5. Correlation analysis between multiple factors and the degumming efficiency of strain HG-49 during ramie degumming. (a) Microbial growth, (b) pectinase activity, (c) xylanase activity, (d) mannanase activity, (e) crystallinity, (f) degree of polymerization. Table S1. List of genes encoding degumming enzymes in the genome of *Pectobacterium carotovorum* HG-49; Table S2. Primers for quantitative real-time PCR analysis. Table S3. Hydrogen bond analysis based on FTIR data of ramie fibers degummed by *Pectobacterium carotovorum* HG-49. Table S4. Average absorbance of ramie fibers in different wavelength. Table S5 The textile parameters of ramie fibers. Table S6. Total RNA purity and concentration of strain HG-49 during ramie degumming; Table S7. Quality statistics of transcriptomic data from strain HG-49 during ramie degumming.
