# Supplementary material for: Degumming of Ramie Bast Fibers by Pectobacterium carotovorum HG-49: Mechanisms and High-Efficiency Strategies
Source: Polymers (Basel). 2026 Jul 20;18(14):1775. doi: 10.3390/polym18141775 (PMC13431327; doi:10.3390/polym18141775)
Supplement: Supplementary file 1 [file polymers-18-01775-s001.zip › polymers-4420417-supplementary.pdf]

Supplementary materials

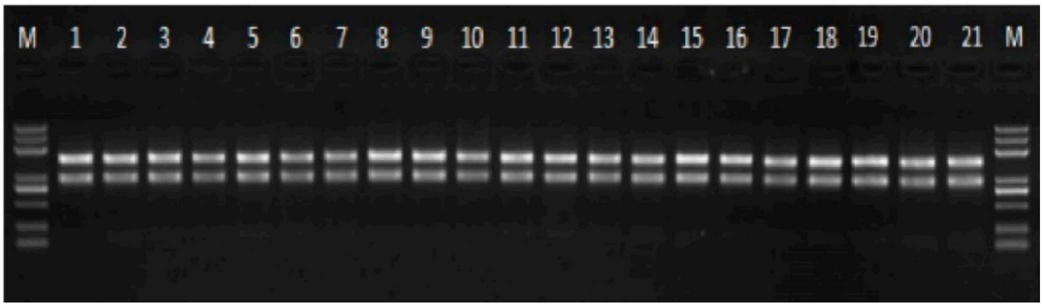

Figure S1. The monosaccharide composition analysis through HPLC detecting.
(a) Standard substance ; (b) Degumming liquids at time 10 h

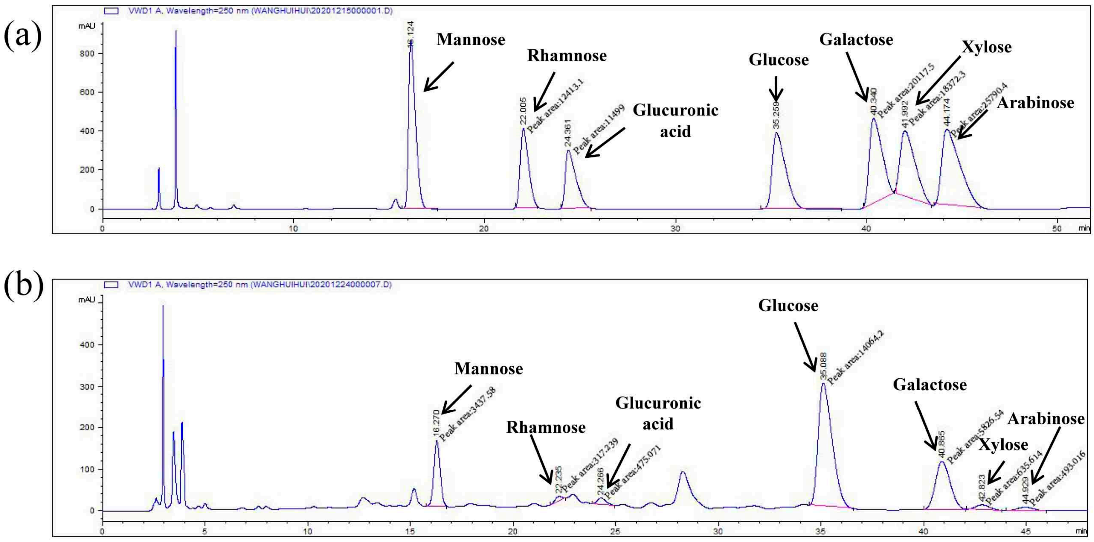

Figure S2. Agarose gel electrophoresis analysis of total RNA extracted from strain HG-49 at different time points of the degumming process. (1–21: three biological replicates at 2 h intervals from 4 to 16 h of degumming, respectively)

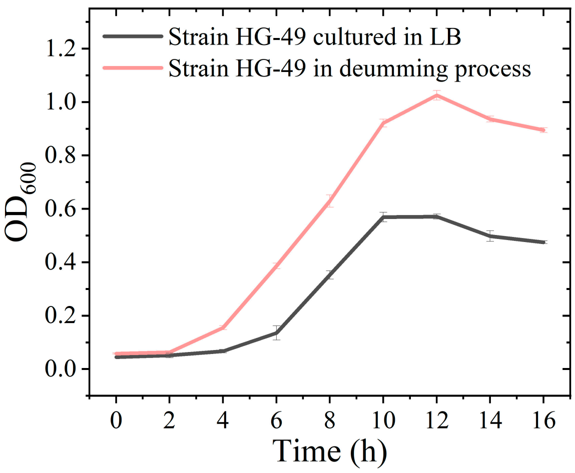

Figure S3. Comparison of growth curves of *Pectobacterium carotovorum* HG-49 in LB medium and during ramie degumming process.

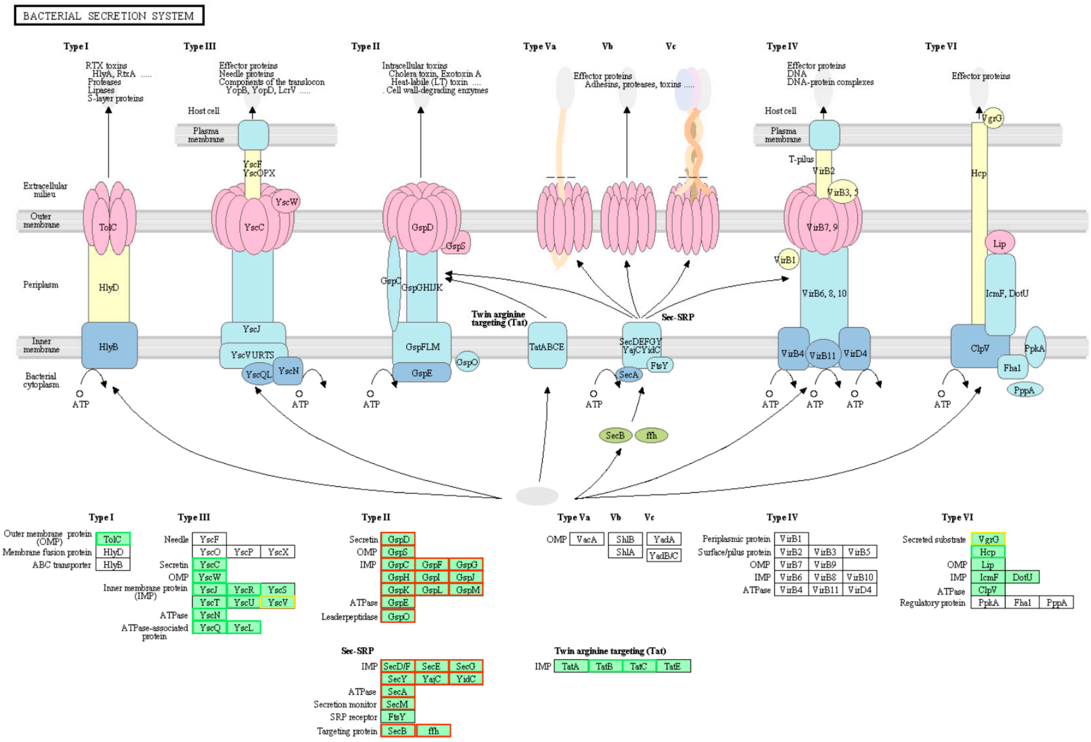

Figure S4. Transcriptomic KEGG pathway analysis comparing *Pectobacterium carotovorum* HG-49 at 4 h versus 12 h of ramie degumming. (Genes in red boxes: upregulated expression, Genes in green boxes: downregulated expression, Gnes in black boxes: absent from strain HG-49)

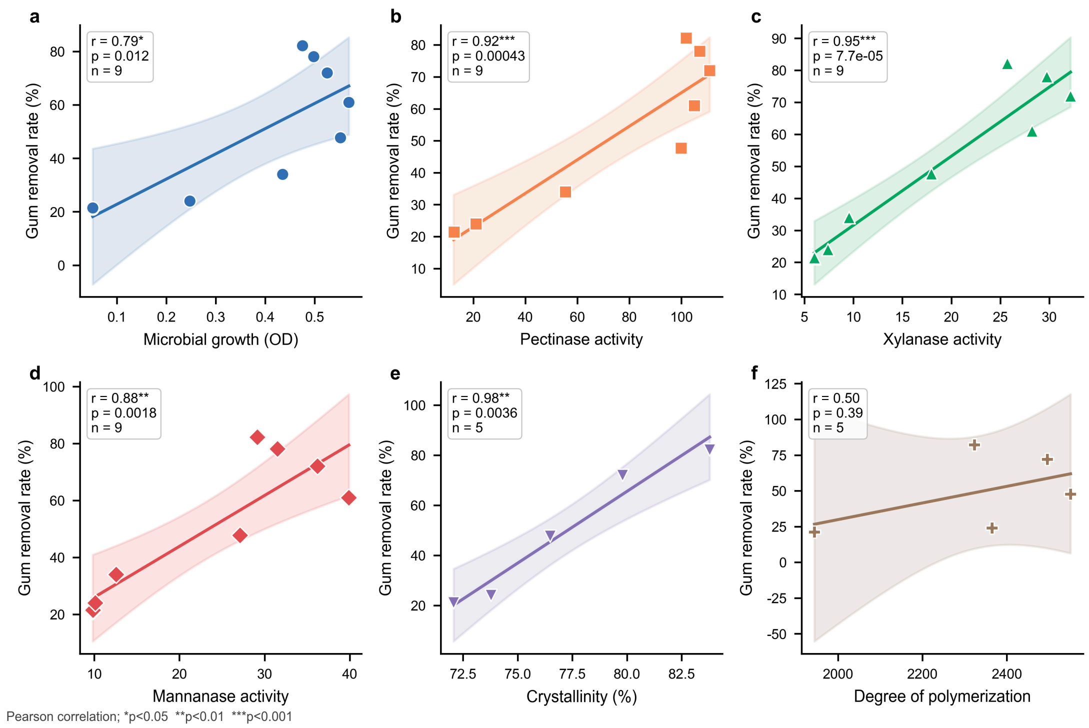

Figure S5. Correlation analysis between multiple factors and the degumming efficiency of strain HG-49 during ramie degumming. (a) Microbial growth, (b) Pectinase activity, (c) Xylanase activity, (d) Mannanase activity, (e) Crystallinity, (f) Degree of polymerization

Table S1. List of genes encoding degumming enzymes in the genome of *Pectobacterium carotovorum* HG-49

Gene ID	DNA chain	Start	End	Length（bp）	Functional description
0368	—	391073	392434	1362	Pectate lyase
0901	—	954468	956036	1569	Pectate lyase
1834	+	1984689	1985813	1125	Pectate lyase
1835	+	1986474	1987598	1125	Pectate lyase
1836	+	1988219	1989343	1125	Pectate lyase
1837	+	1989512	1990807	1296	Pectate lyase
3303	—	3646819	3647916	1098	Pectate lyase
3675	+	4085180	4086124	945	Pectate lyase
4521	—	4934679	4936148	1470	Pectate lyase
0233	+	236337	237968	1632	Pectate disaccharide-lyase
2290	+	2513782	2516016	2235	Pectate disaccharide-lyase
0239	+	243575	244537	963	Pectin acetylesterase
1049	+	1112314	1113420	1107	Pectinesterase

2412	−	2660444	2661634	1191	Pectinesterase
0019	+	15013	16326	1314	Polygalacturonase
0900	−	952362	954341	1980	Polygalacturonase
1326	+	1442208	1443542	1335	Polygalacturonase
3304	+	3648725	3649933	1209	Polygalacturonase
3404	+	3781398	3782753	1356	Polygalacturonase
0257	−	262365	263531	1167	Oligogalacturonide lyase
3558	+	3947022	3948314	1293	Oligogalacturonide lyase
3046	+	3369084	3370781	1698	Rhamnogalacturonate lyase
1586	−	1725091	1726713	1623	Beta-xylosidase
3064	−	3395089	3396657	1569	Beta-xylosidase

Table S2. Primers for quantitative real-time PCR analysis

Primers	Sequence (5'-3')
368 F	CACGGGTTTGTTTATCCAGC
368 R	CGAAGCCATCATCCGAGTTT
1834 F	TCGATTGTAGACGCCGGTTACCTCG
1834 R	CGACAACGTTGGAAGAGTTTACAAT
1835 F	TAGTGGTTTGGCCGTCTGAAGAGG
1835 R	TGGGGTGTATTTATAAGAAATGGA
1836 F	TGGTTGCAGTGGTCAAGACCGGAC
1836 R	AGCGAAGTCAGCCGGGCTCATAAC
1837 F	CGTCAAAGGCGAGTGGGAAGG
1837 R	TTGGCATCGTAAGCAAGGTTATCTG
3303 F	AGTTCAGAATGTAAAGCGGGAGC
3303 R	CCAGCGTGATAACGGGAGG
3675 F	GGCTTATCCAACAACGAATC
3675 R	ACCACCGAAAGAACCAACA
1586 F	CGACGCCAAAGGAACGAAA
1586 R	GATTGCCGCCAGACATAAAGAT
3064 F	GACAGTGGCAATGGCAAGCA
3064 R	GGTACGTCATACCAGGAAGGTTCA

368-3064: Gene IDs of degumming enzymes in strain HG-49

Table S3. Hydrogen bond analysis based on FTIR data of ramie fibers degummed by *Pectobacterium carotovorum* HG-49

Hydrogen bond types	Peak labels	Wavenumber (cm^{-1})	a (%)	b (%)	c (%)	d (%)	e (%)	f (%)
Free OH groups	6	3543	19.15	19.03	18.37	17.38	17.04	16.28
Intermolecular hydrogen bond	1, 5	3089, 3508	43.95	43.53	43.01	42.77	42.51	42.05
Intramoleculat hydrogen bond	2, 3, 4	3216, 3290, 3309	36.90	37.44	38.62	39.85	40.45	41.67

a: Ramie bast fibers, b: Ramie fibers degummed for 4 h, c: Ramie fibers degummed for 8 h, d: Ramie fibers degummed for 12 h, e: Ramie fibers degummed for 16 h, f: Refined ramie fibers

Table S4. Average absorbance of ramie fibers in different wavelength

Wavelength	1372 cm^{-1}	1640 cm^{-1}	1737 cm^{-1}	1512 cm^{-1}
Ramie bast fibers	0.512 ± 0.001	0.081 ± 0.002	0.135 ± 0.002	0.041 ± 0.002
Ramie fibers degummed for 4 h	0.521 ± 0.002	0.076 ± 0.002	0.134 ± 0.003	0.039 ± 0.001
Ramie fibers degummed for 8 h	0.550 ± 0.001	0.055 ± 0.001	0.110 ± 0.001	0.030 ± 0.001
Ramie fibers degummed for 12 h	0.592 ± 0.002	0.021 ± 0.002	0.076 ± 0.002	0.019 ± 0.001
Ramie fibers degummed for 16 h	0.614 ± 0.002	0.009 ± 0.002	0.045 ± 0.001	0.016 ± 0.001
Refined ramie fibers	0.652 ± 0.003	0.006 ± 0.001	0.015 ± 0.001	0.005 ± 0.001

Table S5. The textile parameters of ramie fibers

Textile parameters	Residual gum content (%)	Whiteness (%)	Bundle breaking tenacity (cN/dtex)
Degummed ramie fibers (16 h)	6.65 ± 0.26	41.2 ± 0.5	4. 76 ± 0.09
Refined ramie fibers	1.98 ± 0.14	57.5 ± 1.1	4.95 ± 0.11

Table S6. Total RNA purity and concentration of strain HG-49 during ramie degumming

Samples	OD_{260}/OD_{280}	OD_{260}/OD_{230}	Concentration（ng/μL）
1	1.98	2.25	585
2	2.01	2.30	573
3	1.99	2.28	561
4	1.95	2.31	521
5	2.03	2.20	581
6	1.98	2.19	509
7	1.94	2.29	498
8	2.01	2.21	583

9	2.05	2.22	532
10	1.93	2.34	576
11	2.05	2.23	587
12	2.03	2.24	541
13	2.03	2.32	518
14	2.01	2.21	630
15	2.05	2.29	612
16	2.04	2.29	514
17	1.94	2.31	518
18	2.02	2.20	624
19	2.04	2.24	579
20	1.97	2.26	519
21	1.96	2.24	556

1-21: three biological replicates at 2 h intervals from 4 to 16 h of degumming, respectively

Table S7. Quality statistics of transcriptomic data from strain HG-49 during ramie degumming

Sample	Raw sequence	Clean sequence	Total reads	Error rate(%)	Q20 (%)	Q30 (%)	GC (%)	Alignment rate (%)
4a	10353430	10087988	1.51G	0.02	97.66	93.31	50.32	98.85
4b	12668162	12328866	1.85G	0.02	97.65	93.28	50.40	99.43
4c	10623916	10380682	1.56G	0.02	97.53	93.05	50.51	99.03
6a	12326252	12066302	1.81G	0.02	97.59	93.16	50.77	98.85
6b	9428654	9186558	1.38G	0.02	97.54	93.03	50.80	99.61
6c	11947980	11644408	1.75G	0.02	97.39	92.72	50.68	98.99
8a	11399886	11156212	1.67G	0.02	97.71	93.42	50.79	98.9
8b	10174466	9970478	1.5G	0.02	97.61	93.16	50.92	99.45
8c	11598756	11306082	1.7G	0.02	97.47	92.92	50.80	98.8
10a	10371856	10140710	1.52G	0.02	97.45	92.87	50.59	99.06
10b	9912124	9667316	1.45G	0.02	97.60	93.15	50.89	99.4
10c	15532568	15206064	2.28G	0.02	97.31	92.47	50.89	98.95
12a	9141864	8941028	1.34G	0.02	97.64	93.25	50.62	99.23
12b	9098778	8776648	1.32G	0.02	97.52	93.00	50.69	99.03
12c	12117926	11693016	1.75G	0.02	97.33	92.60	50.65	98.62
14a	10032728	9821638	1.47G	0.02	97.23	92.27	50.53	98.93
14b	9480572	9174202	1.38G	0.02	97.62	93.20	50.56	98.78
14c	13376084	12932084	1.94G	0.02	97.40	92.74	50.54	99.12
16a	12748936	12494920	1.87G	0.02	97.07	91.94	50.37	99.15

16b	13417548	13038690	1.96G	0.02	97.29	92.42	50.45	99.22
16c	9998102	9599702	1.44G	0.02	97.59	93.11	50.26	99.38

Q20 and Q30: the percentages of bases with Phred quality scores greater than 20 and 30, respectively, relative to the total number of bases. 4-16 a, 4-16 b, 4-16 c: Three experimental replicates at degumming time 4 h, 6 h, 8 h, 10 h, 12 h, 14 h and 16 h.
